# Blind Equalization Based on Modified Third-Order Moment Algorithm for PAM-PPM Optical Signals in FSO Communication

**DOI:** 10.3390/s25227063

**Published:** 2025-11-19

**Authors:** Shutian Luo, Xiaofeng Li

**Affiliations:** School of Aeronautics and Astronautics, University of Electronic Science and Technology of China, Chengdu 611731, China; shutianluo@std.uestc.edu.cn

**Keywords:** PAM-PPM optical signal, blind equalization, modified third-order moment algorithm, FSO communication

## Abstract

In order to mitigate the influence of turbulence on pulse amplitude modulation–pulse position modulation (PAM-PPM) optical signals, which represents a promising avenue for future high-speed free-space optical (FSO) communication, this paper proposes a novel blind equalization scheme based on a modified third-order moment algorithm (MTOMA). The MTOMA is more robust to noise compared with the current fourth-order moment algorithms, such as the constant modulus algorithm (CMA) and the modified constant modulus algorithm (MCMA). Moreover, it will not increase the implementation complexity compared with the CMA and MCMA. The simulation results show that the MTOMA effectively reduces the distortion of PAM-PPM optical signals in atmospheric turbulence channels with a pointing error. Under different turbulence conditions, the MTOMA has a faster convergence rate than the CMA and MCMA. For example, when the signal-to-noise ratio (SNR) is 15 dB, the MTOMA requires about 530 iterations to reach convergence in moderate turbulence, which is about 230 and 170 fewer iterations than required by the CMA and MCMA, respectively; in addition, the differences in the number of iterations required by the MTOMA and those required by the CMA and MCMA, respectively, are 140 and 100 in weak turbulence and 150 and 90 in strong turbulence. Moreover, when the algorithms converge, the bit error rate (BER) performance of the PAM-PPM signals with MTOMA is also superior to that with CMA and MCMA. For example, when SNR = 20 dB, the BER performance of the PAM-PPM signals with MTOMA improves by 6.5 dB and 1.7 dB, respectively, compared to that with CMA and MCMA in moderate turbulence; this value improves by 4.3 dB and 1.4 dB in weak turbulence and 4.8 dB and 1.5 dB in strong turbulence. In addition, when the MTOMA reaches convergence, the decision-directed least mean square (DDLMS) algorithm can continue to be utilized to further improve the BER performance of PAM-PPM optical signals.

## 1. Introduction

The rapid development of various applications of wireless communication has led to the requirement for ultrahigh-speed data transmission [1]. The transmission rate of traditional microwave communication is insufficient to support the tremendous amount of data being transmitted, so novel wireless transmission technologies are urgently needed [2,3]. With its abundant bandwidth, fast transmission rate, good flexibility, and immunity to electromagnetic interference, free-space optical (FSO) communication is promising in terms of future wireless applications and has attracted substantial attention from researchers [1,2,3,4,5]. Moreover, it has become the focus of research on sixth-generation (6G) technologies, which will further promote its development [6].

Modulation is one of the core technologies in FSO communication, with the aim of loading information onto an optical carrier [7], and contains intensity modulation–direct detection (IM-DD) schemes and coherent detection schemes. At present, IM-DD schemes are mainly used in practical applications, because they have a simpler structure and lower implementation complexity [8]. IM-DD schemes include on–off keying (OOK), pulse position modulation (PPM), subcarrier intensity modulation (SIM), etc. [9,10,11]. However, to perform optimally, OOK requires an adaptive threshold, and SIM has a complex structure, which can increase their implementation costs [9,10]. By contrast, PPM has potential because it balances implementation complexity and transmission performance, and also has high power efficiency, making it suitable for long-distance communication [11].

However, when an optical signal propagates in atmospheric turbulence, it can be subjected to the turbulence effect, which is caused by irregular changes in atmospheric properties such as speed, temperature, pressure, and refractive index [12]. The turbulence effect can induce aberrations in optical signals, such as intensity fluctuation and phase distortion [12]. IM-DD systems utilize intensity to carry information, so intensity fluctuation is the primary factor affecting their performance [12]. In microwave communication, equalization technology, a kind of digital signal processing (DSP) technique, is often utilized to reduce signal distortion because it can easily be integrated into the receiver [13]. Generally, it uses a finite impulse response (FIR) filter to compensate for distortion and obtain the improved signals [13]. Inspired by equalization technology, equalization algorithms have been utilized in IM-DD systems to mitigate the turbulence effect [14,15], and they have also been applied to PPM signals [16,17,18,19].

Ref. [16] utilized the Viterbi equalization algorithm to improve the bit error rate (BER) performance of PPM optical signals in a turbulence channel, but the calculation formulas are quite complicated. Ref. [17] utilized the minimum mean square error (MMSE) algorithm based on a training sequence to improve the transmission performance of PPM signals in FSO systems, but the training sequence reduced the transmission efficiency. Ref. [18] utilized the decision-directed least mean square (DD-LMS) algorithm to reduce the distortion of PPM signals, but the convergence rate of DD-LMS is slow, and it cannot rapidly track the channel status. Ref. [19] utilized constant modulus algorithm (CMA) equalization to mitigate the influence of the turbulence effect on PPM optical signals. It has a fast convergence rate and low implementation complexity, and does not need a training sequence. Meanwhile, it was found that, although PPM is not a constant modulus signal, the CMA can still improve its BER performance.

FSO communication is advancing in the direction of high-speed transmission, which is generally achieved by increasing spectral efficiency [20]. However, the spectral efficiency of a PPM signal will decrease with an increase in the modulation order, so the traditional PPM format is not suitable for high-speed FSO systems [21]. In order to improve PPM’s spectral efficiency, the multi-amplitude PPM format is proposed [22], which is also known as pulse amplitude modulation—PPM (PAM-PPM). It can achieve a faster transmission rate than the traditional PPM format and better meet the needs of future high-speed FSO systems [22].

However, a PAM-PPM optical signal is also limited in the face of high-speed transmission. Due to the turbulence effect, the refractive index of the atmosphere exhibits spatial inhomogeneity [23]. In this case, an optical pulse can be regarded as a combination of several sub-pulses, and they can arrive at the receiver at different times [24]. As the transmission speed increases, the pulse width of the PAM-PPM signal can become shorter in the time domain. Thus, in the time domain, the ratio of the inter-arrival time of sub-pulses to the pulse width can become larger, and then the overlap between sub-pulses can be reduced [24]. In this situation, the intensity fluctuation of optical pulses will be more severe at the receiver, and the equalization algorithms will require more iterations to reach convergence [24]. Therefore, it can be deduced that, for high-speed PAM-PPM optical signals, the system reliability will decrease with current CMA equalization. In order to improve the reliability of high-speed PAM-PPM optical systems in a turbulence channel, it is necessary to explore novel equalization algorithms that have faster convergence rates than the CMA.

Ref. [25] demonstrated that the third-order moment algorithm (TOMA) used in the blind equalization process has convergence properties when processing asymmetric signals, and it has a faster convergence rate than fourth-order moment algorithms, such as the CMA. A PAM-PPM signal is actually an asymmetric signal, which will be proven in Section 2. Thus, according to Ref. [25], the TOMA used in the blind equalization process can be convergent when processing a PAM-PPM signal. However, we find that the PAM-PPM signal cannot be restored when TOMA reaches convergence. Therefore, on the basis of TOMA, we propose using a modified third-order moment algorithm (MTOMA) as a blind equalization scheme, which is able to restore the PAM-PPM signal under the convergence state, and the results are compared with the fourth-order moment algorithms.

In addition, for FSO communication, a pointing error will occur when the transmitter and receiver are not aligned. This can induce power attenuation at the receiver, affecting the transmission performance of the system [26,27]. Therefore, the pointing error is also considered in the atmospheric channel.

This paper is organized as follows. In Section 2, the asymmetric characteristic of the PAM-PPM signal is proven, and the channel model combining atmospheric turbulence and the pointing error is established. In Section 3, the principles of the MTOMA are introduced, and its performance is analyzed. In Section 4, the simulations are conducted and discussed, and in Section 5, the conclusions are drawn.

## 2. System Modeling

### 2.1. Block Diagrams of Transmitter and Receiver

If the order of PAM is *m* and the order of PPM is *l*, the signal can be abbreviated to *m*PAM-*l*PPM. Each symbol consists of ${log}_{2}m+{log}_{2}l$ bits and is divided into *l* parts in the time domain, which are called the time slots. The pulse occupies one slot, and its amplitude has *m* values.

**Figure 1:**
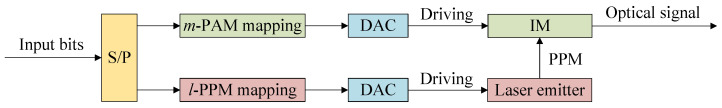
Block diagram of transmitter. S/P: serial-to-parallel; DAC: digital-to-analog converter; IM: intensity modulator.

**Figure 2:**
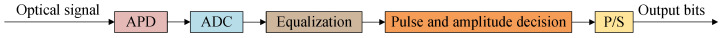
Block diagram of receiver. APD: avalanche photon diode; ADC: analog-to-digital converter; P/S: parallel-to-serial.

Block diagrams of the transmitter and receiver of *m*PAM-*l*PPM are presented in Figure 1 and Figure 2, respectively. At the transmitter, the serial binary sequence will be converted into the *m*PAM-*l*PPM symbols through serial-to-parallel (S/P) conversion. For each symbol, suppose that the first ${log}_{2}l$ bits will be mapped to generate the *l*-PPM driving signal, and the remaining ${log}_{2}m$ bits will be mapped to generate the *m*-PAM driving signal, in the discrete-time case. The process is completed through DSP. Then, the driving signals will be converted into analog waves by a digital-to-analog converter (DAC). The PPM driving wave can drive the laser emitter to emit the *l*-PPM optical signal. And the *m*-PAM driving wave can drive the intensity modulator (IM), such as a Mach–Zehnder modulator, to change the laser intensity. Finally, the *m*PAM-*l*PPM optical signal will be transmitted into the atmospheric channel.

At the receiver, firstly, the *m*PAM-*l*PPM optical signal is received and directly detected by the avalanche photon diode (APD), which can convert optical signals into electrical signals. Each time slot of the *m*PAM-*l*PPM electrical signal will be sampled by the analog-to-digital converter (ADC), and we suppose that the sampling has satisfied the symbol synchronization and slot synchronization. Next, the discrete signals will be processed by the equalization module to reduce the distortion induced by turbulence effects, and the output signals will be used for symbol decisions. For each symbol, there are *l* sampling values, and the slot with the maximum sampling value will be considered the pulse slot. Thus, the first ${log}_{2}l$ bits can be restored. Then, the sampling value of the pulse slot will be further determined to restore the remaining ${log}_{2}m$ bits according to the decision thresholds. Finally, the output serial binary sequence can be obtained through parallel-to-serial (P/S) conversion.

### 2.2. Proof of Asymmetric Characteristic of PAM-PPM Signal

The discrete *m*PAM-*l*PPM signal can be written as

(1) $$s\left[n\right]=\sum_{k=0}^{\infty }A_{k}\delta \left[n-kT_{s}-c_{k}T_{\mathrm{slot}}\right]$$

where $\delta \left[n\right]$ is the impulse signal in the discrete-time case; $A_{k}$ is an amplitude parameter with *m* values (which we assume to be 2, 4, 6, …, 2m−2, 2m (m>1) in this paper); $T_{s}$ and $T_{\mathrm{slot}}$ are symbol and slot intervals, respectively; and $c_{k}$ is the position parameter (with values of 0, 1, …, l−1), which determines the specific position of pulses.

The signal with non-zero skewness is called the asymmetric signal [25]. The skewness of the *m*PAM-*l*PPM signal can be expressed as

(2) $$S\left(s\right)=\frac{E\left[{\left(s-\mu \right)}^{3}\right]}{{\left(\sigma^{2}\right)}^{3/2}}$$

where μ and $\sigma^{2}$ are the mean and variance of the *m*PAM-*l*PPM signal, respectively. Thus, if the value of $E[{\left(s-\mu \right)}^{3}]$ is not equal to zero, the signal can be regarded as an asymmetric signal.

$E[{\left(s-\mu \right)}^{3}]$ can be written as $E\left[{\left(s-\mu \right)}^{3}\right]=E\left(s^{3}-3s^{2}\mu +3s\mu^{2}-\mu^{3}\right)$. Suppose that the prior probabilities of each symbol are identical; then, μ can be derived as

(3) $$\mu =E(s)=\frac{1}{m}\cdot \frac{1}{l}\left(2+4+\ldots +2m\right)=\frac{m+1}{l}.$$

$E\left(s^{2}\right)$ can be derived as

(4) $$E\left(s^{2}\right)=\frac{1}{m}\cdot \frac{1}{l}\left[2^{2}+4^{2}+\ldots +{\left(2m\right)}^{2}\right]=\frac{2\left(m+1\right)\left(2m+1\right)}{3l}.$$

$E\left(s^{3}\right)$ can be derived as

(5) $$E\left(s^{3}\right)=\frac{1}{m}\cdot \frac{1}{l}\left[2^{3}+4^{3}+\ldots +{\left(2m\right)}^{3}\right]=\frac{2m{\left(m+1\right)}^{2}}{l}.$$

Thus, $E[{\left(s-\mu \right)}^{3}]$ can be derived as

(6) $$E\left[{\left(s-\mu \right)}^{3}\right]=\frac{2{\left(m+1\right)}^{2}\left(ml^{2}-2ml-l+m+1\right)}{l^{3}}.$$

In Equation (6), $E[{\left(s-\mu \right)}^{3}]=0$ means $ml^{2}-2ml-l+m+1=0$; in other words, $m{\left(l-1\right)}^{2}=l-1$. *l* is a positive integer greater than 1, so if m>1, it can be derived that $m{\left(l-1\right)}^{2}\neq l-1$; in other words, $E[{\left(s-\mu \right)}^{3}]\neq 0$. Therefore, when m>1 and l>1, the *m*PAM-*l*PPM signal has an asymmetric characteristic.

### 2.3. Signal Transmission in Turbulence Channel with Pointing Error

In the continuous-time case, the *m*PAM-*l*PPM signal can be written as

(7) $$m\left(t\right)=p\left(t\right)*\sum_{k=0}^{\infty }A_{k}\delta \left(t-kT_{s}-c_{k}T_{\mathrm{slot}}\right)=\sum_{k=0}^{\infty }A_{k}p\left(t-kT_{s}-c_{k}T_{\mathrm{slot}}\right)$$

where $p\left(t\right)$ denotes the Nyquist pulse shaping, and the maximum amplitude of $p\left(t\right)$ is 1; $\delta \left(t\right)$ is the impulse signal in the continuous-time case; and the symbol “∗” denotes convolution.

Suppose that the intensity of the optical carrier is *I*. At the transmitter, the envelope of the *m*PAM-*l*PPM optical signal is given by

(8) $$s\left(t\right)={\left[Im\left(t\right)\right]}^{1/2}={\left[I\sum_{k=0}^{\infty }A_{k}p\left(t-kT_{s}-c_{k}T_{\mathrm{slot}}\right)\right]}^{1/2}.$$

At the receiver, the optical signal can be directly converted into an electrical signal by the APD, which can be written as

(9) $$r\left(t\right)=\eta {\left[s\left(t\right)\right]}^{2}+n\left(t\right)=\eta I\sum_{k=0}^{\infty }A_{k}p\left(t-kT_{s}-c_{k}T_{\mathrm{slot}}\right)+n\left(t\right)$$

where η is the optical-to-electrical conversion efficiency. In FSO communication, $n\left(t\right)$ can represent additive white Gaussian noise (AWGN) with a mean of 0 and variance of $\sigma_{n}^{2}$ [28].

The received laser intensity *I* can be written as $I=I_{A}I_{P}$, where $I_{A}$ denotes the intensity fluctuation induced by atmospheric turbulence, and $I_{P}$ denotes the intensity attenuation induced by the pointing error [29].

The commonly used turbulence models are the Log-normal, Gamma–Gamma, and Málaga models [30]. The Gamma–Gamma and Málaga distributions are the most appropriate models to represent conditions ranging from weak to strong turbulence, whereas the Log-normal model is primarily useful for weak turbulence [30]. Moreover, the Málaga distribution is more complex than Gamma–Gamma distribution and can involve complicated calculations. Therefore, the Gamma–Gamma turbulence model is adopted in this paper. Under the normalized power condition, $E\left(I_{A}\right)=1$, and the probability density function (PDF) of $I_{A}$ is given by [29]

(10) $$f_{I_{A}}\left(i_{a}\right)=\frac{2}{\Gamma \left(\alpha \right)\Gamma \left(\beta \right)}{\left(\alpha \beta \right)}^{\frac{\alpha +\beta }{2}}{i_{a}}^{\frac{\alpha +\beta }{2}-1}K_{\alpha -\beta }\left(2\sqrt{\alpha \beta i_{a}}\right).$$

where $\Gamma \left(\cdot \right)$ is the Gamma function, $K_{v}\left(\cdot \right)$ is a modified Bessel function of the second kind, and α and β are the parameters related to atmospheric turbulence, which are given by [29]

(11) $$\alpha ={\left\{\exp\left[\frac{0.49\sigma_{R}^{2}}{{\left(1+1.11\sigma_{R}^{12/5}\right)}^{7/6}}\right]-1\right\}}^{-1}$$

(12) $$\beta ={\left\{\exp\left[\frac{0.51\sigma_{R}^{2}}{{\left(1+0.69\sigma_{R}^{12/5}\right)}^{5/6}}\right]-1\right\}}^{-1}.$$

Here, $\sigma_{R}^{2}$ is the Rytov variance, and $\sigma_{R}^{2}=1.23C_{n}^{2}k^{7/6}z^{11/6}$, where $C_{n}^{2}$ is the refractive-index structure parameter, *k* is the wave number, and *z* is the propagation distance [29]. Generally, $\sigma_{R}^{2}<1$ represents weak turbulence, $\sigma_{R}^{2}∼1$ represents moderate turbulence, and $\sigma_{R}^{2}>1$ represents strong turbulence [29].

**Figure 3:**
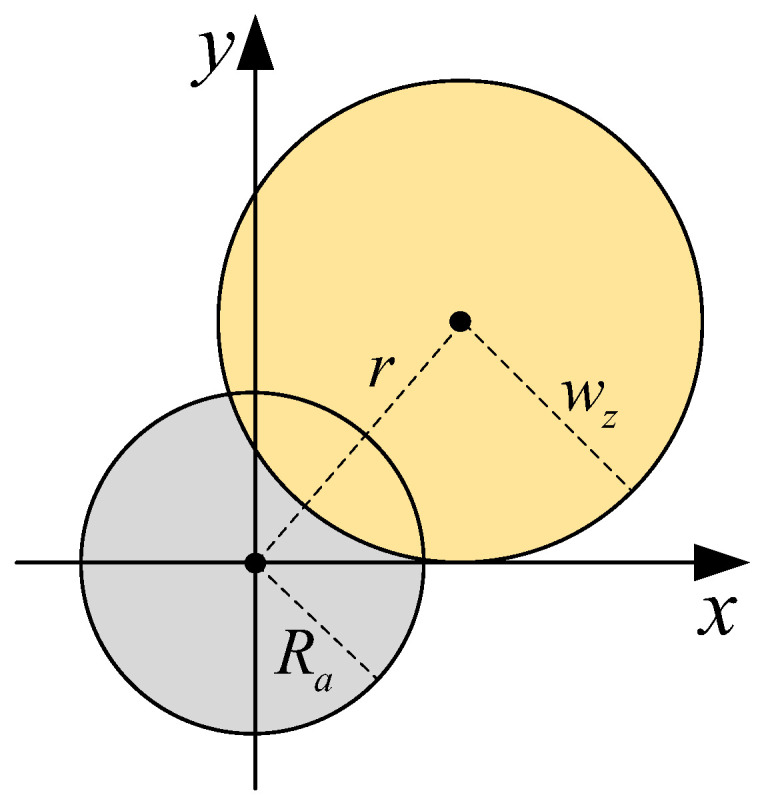
Detector and beam footprint with misalignment on the detector plane.

At the receiver, a pointing error can cause misalignment between the beam center and detector center, as shown in Figure 3. Ref. [31] proposes a novel pointing error model named the modified intensity uniform model, which has better approximation accuracy and simpler expression than the previous Farid model. It is expressed as [31]

(13) $$I_{P}\left(r;z\right)=\delta \exp\left(-\delta \frac{r^{2}}{R_{a}^{2}}\right).$$

$I_{P}\left(\cdot \right)$ represents the fraction of the power collected by the detector; *r* is the distance between the beam center and detector center; $R_{a}$ is the radius of the detector; and δ is given by $\delta =1-\exp\left(-2R_{a}^{2}/w_{z}^{2}\right)$, where $w_{z}=w_{0}\sqrt{1+\varepsilon {\left(\lambda z/\pi {w_{0}}^{2}\right)}^{2}}$ is the beam radius at distance *z* and $w_{0}$ is the beam radius at z=0, λ is the wavelength, $\varepsilon =\left[1+2w_{0}^{2}/\rho_{0}^{2}\left(z\right)\right]$, and $\rho_{0}={\left(0.55C_{n}^{2}k^{2}z\right)}^{-3/5}$ [31].

The parameter *r* follows a Rayleigh distribution, which is given by [27]

(14) $$\begin{array}{cc} f_{r}\left(r\right)=\frac{r}{\sigma_{s}^{2}}\exp\left(-\frac{r^{2}}{2\sigma_{s}^{2}}\right), & r>0 \end{array}$$

where $\sigma_{s}$ is the displacement standard deviation at the receiver.

Combining Equations (13) and (14), the PDF of $I_{P}$ can be derived as

(15) $$f_{I_{P}}\left(i_{p}\right)=\begin{array}{cc} \frac{\gamma }{\delta^{\gamma }}{i_{p}}^{\gamma -1}, & 0\leq i_{p}\leq \delta \end{array}$$

where $\gamma =R_{a}^{2}/2\delta \sigma_{s}^{2}$.

When $I_{A}=i_{a}$, the cumulative distribution function (CDF) of received laser intensity *I* can be expressed as

(16) $$F_{I}\left(i\left|I_{A}=i_{a}\right.\right)=P\left(I_{P}\leq \frac{i}{i_{a}}\right)=F_{I_{P}}\left(\frac{i}{i_{a}}\right)$$

where $F_{I_{P}}\left(\cdot \right)$ is the CDF of $I_{P}$.

The conditional PDF of *I* can be derived as

(17) $$f_{I}\left(i\left|I_{A}=i_{a}\right.\right)=\frac{1}{i_{a}}f_{I_{P}}\left(\frac{i}{i_{a}}\right).$$

The PDF of *I* can be expressed as

(18) $$f_{I}\left(i\right)=\int_{i/\delta }^{+\infty }f_{I}\left(i\left|I_{A}=i_{a}\right.\right)f_{I_{A}}\left(i_{a}\right)di_{a}.$$

Combining Equations (10), (15), (17), and (18), it can be determined that

(19) $$f_{I}\left(i\right)=\frac{2\gamma {\left(\alpha \beta \right)}^{\frac{\alpha +\beta }{2}}}{\delta^{\gamma }\Gamma \left(\alpha \right)\Gamma \left(\beta \right)}\int_{i/\delta }^{+\infty }i^{\gamma -1}{i_{a}}^{\frac{\alpha +\beta }{2}-\gamma -1}K_{\alpha -\beta }\left(2\sqrt{\alpha \beta i_{a}}\right)di_{a}.$$

## 3. Blind Equalization Scheme Based on Modified Third-Order Moment Algorithm

### 3.1. Iterative Formula of the Algorithm

**Figure 4:**
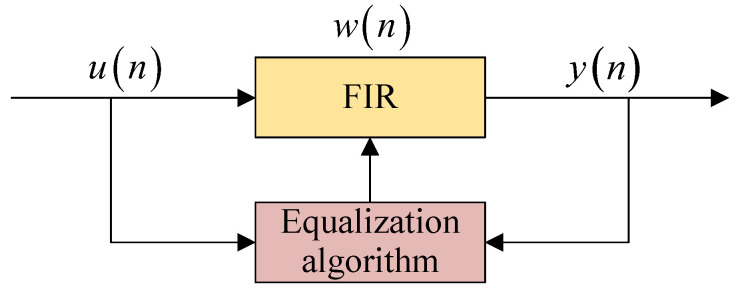
Block diagram of blind equalization process. $u\left(n\right)$: input vector; $w\left(n\right)$: coefficient vector of finite impulse response (FIR) filter; $y\left(n\right)$: output signal.

A block diagram of the blind equalization process is shown in Figure 4, where *n* denotes the *n*-th iteration [32]. The output signal $y\left(n\right)=u^{T}\left(n\right)w\left(n\right)$ can be obtained when the input vector $u\left(n\right)$ passes through the finite impulse response (FIR) filter, whose coefficient vector is $w\left(n\right)$. $w\left(n\right)$ can be updated adaptively by the equalization algorithm. The updated $w\left(n+1\right)$ based on TOMA is given by [25]

(20) $$w\left(n+1\right)=w\left(n\right)+\mu \nabla J$$

where μ is the step size and *J* is the objective function.

*J* can be expressed as $E({\left|y\left(n\right)\right|}^{3})$, and our analysis indicates that TOMA equalization cannot restore the *m*PAM-*l*PPM signal under a convergence state, the reason for this is as follows:

The derivative ∇J can be derived as

(21) $$\nabla J=\frac{\partial J}{\partial w\left(n\right)}=3E\left({\left|y\left(n\right)\right|}^{2}\right)\nabla \left|y\left(n\right)\right|.$$

The $y\left(n\right)$ of the *m*PAM-*l*PPM signal is a real number, so $\nabla \left({\left|y\left(n\right)\right|}^{2}\right)=\nabla \left(y^{2}\left(n\right)\right)$, and we can also obtain $2\left|y\left(n\right)\right|\nabla \left|y\left(n\right)\right|=2y\left(n\right)\nabla y\left(n\right)$. Because $\nabla y\left(n\right)=u\left(n\right)$, $\nabla \left|y\left(n\right)\right|$ can be represented as $\nabla \left|y\left(n\right)\right|=u\left(n\right)y\left(n\right){\left|y\left(n\right)\right|}^{-1}$. Combining this with Equations (20) and (21) while replacing average values with instantaneous values, it can be determined that

(22) $$w\left(n+1\right)=w\left(n\right)+\mu u\left(n\right)y\left(n\right)\left|y\left(n\right)\right|.$$

Ignoring the impact of noise, when the TOMA reaches convergence, ∇J is equal to zero; in other words, $u\left(n\right)y\left(n\right)\left|y\left(n\right)\right|$ in Equation (22) is equal to zero. However, in this situation, the output $y\left(n\right)$ must always be zero, and the signal cannot be restored. Therefore, TOMA equalization is not suitable for the *m*PAM-*l*PPM signal.

Inspired by the Godard algorithms, in order to restore the *m*PAM-*l*PPM signal, Equation (22) can be modified as follows:

(23) $$w\left(n+1\right)=w\left(n\right)+\mu u\left(n\right)y\left(n\right)\left[\left|y\left(n\right)\right|-R\right]$$

where *R* is a real parameter.

According to the Godard algorithms, as the iteration number increases, the appropriate parameter *R* can not only extract the expected output signal, but also make the iterative formula convergent [33]. The Godard algorithms generally process the constant modulus signals, so the parameter *R* is usually fixed. However, *m*PAM-*l*PPM is not a constant modulus signal, and turbulence channels can change rapidly. Under these circumstances, the performance of equalization algorithms with fixed *R* will be significantly restricted [34]. In this situation, one effective method is to adjust *R* adaptively based on the variation in the received signals, which can also ensure the convergence of equalization algorithms and reduce the steady-state error induced by noise [34].

Therefore, for the *m*PAM-*l*PPM signal, Equation (23) can be further modified as follows:

(24) $$w\left(n+1\right)=w\left(n\right)+\mu u\left(n\right)y\left(n\right)\left[\left|y\left(n\right)\right|-R\left(n\right)\right]$$

where $R\left(n\right)$ is a variable parameter. We define Equation (24) as the modified third-order moment algorithm (MTOMA).

In Section 2, it is assumed that the pulse amplitudes of *m*PAM-*l*PPM are 2, 4, 6, …, 2m−2, 2m (m>1) at the transmitter. However, the amplitudes can be attenuated by pointing errors, and the average values of pulse amplitudes at the receiver can be attenuated to $2\eta E\left(I\right)$, $4\eta E\left(I\right)$, $6\eta E\left(I\right)$, …, $\left(2m-2\right)\eta E\left(I\right)$, $2m\eta E\left(I\right)$ (m>1). In this case, according to Ref. [34], we propose the variation principle of $R\left(n\right)$, as shown in Table 1. If $y\left(n\right)\leq \eta E\left(I\right)$, $R\left(n\right)$ is set to 0; if (2k−1)ηE(I)<y(n)≤(2k+1)ηE(I), $R\left(n\right)$ is set to 2kηE(I), k=1,2,…,m−1; if $y\left(n\right)>\left(2m-1\right)\eta E\left(I\right)$, $R\left(n\right)$ is set to $2m\eta E\left(I\right)$. It can be seen that $R\left(n\right)$ can vary adaptively according to the values of $y\left(n\right)$.

**Figure 5:**
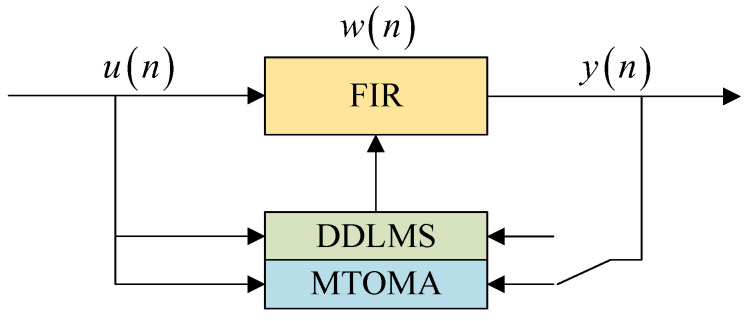
Block diagram of MTOMA-DDLMS dual-mode equalization.

When the CMA converges, the steady-state error can be further reduced by continuing to use the DDLMS algorithm, which can achieve a low steady-state error [35]. Inspired by this, we continue to use the DDLMS algorithm when the MTOMA reaches convergence; we call this dual-mode algorithm the MTOMA-DDLMS, a block diagram of which is shown in Figure 5.

The iterative formula of the DDLMS algorithm is given by [36]

(25) $$w_{D}\left(n+1\right)=w_{D}\left(n\right)+\mu u\left(n\right)e_{D}\left(n\right).$$

Here, $e_{D}\left(n\right)$ is the error function, which can be written as [36]

(26) $$e_{D}\left(n\right)=dec\left[y\left(n\right)\right]-y\left(n\right)$$

where $dec\left(\cdot \right)$ is the decision function.

Similarly to the decision principle of $R\left(n\right)$, $dec\left(\cdot \right)$ can be defined as

(27) $$dec\left[y(n)\right]=\left\{\begin{array}{c} 0\\ 2k\eta E(I)\\ 2m\eta E(I) \end{array}\right.\begin{array}{c} y(n)\leq \eta E(I)\\ \left(2k-1)\eta E(I)<y(n)\leq (2k+1)\eta E(I\right)\\ y(n)>(2m-1)\eta E(I) \end{array}$$

where k=1,2,…,m−1.

### 3.2. Performance Analysis and Comparison

Inspired by the analysis above, we also make the originally fixed dispersion constant $R_{2}$ in the CMA variable, which can be expressed as $R_{2}\left(n\right)$; in this paper, this algorithm is defined as the modified constant modulus algorithm (MCMA). Specifically, for *m*PAM-*l*PPM, if ${\left|y\left(n\right)\right|}^{2}\leq {\left[\eta E\left(I\right)\right]}^{2}$, $R_{2}\left(n\right)=0$; if ${\left[\left(2k-1\right)\eta E\left(I\right)\right]}^{2}<{\left|y\left(n\right)\right|}^{2}\leq {\left[\left(2k+1\right)\eta E\left(I\right)\right]}^{2}$, $R_{2}\left(n\right)={\left[2k\eta E\left(I\right)\right]}^{2}$, k=1,2,…,m−1; and if ${\left|y\left(n\right)\right|}^{2}>{\left[\left(2m-1\right)\eta E\left(I\right)\right]}^{2}$, $R_{2}\left(n\right)={\left[2m\eta E\left(I\right)\right]}^{2}$.

#### 3.2.1. Robustness to Noise

The CMA and MCMA are fourth-order moment algorithms, while the MTOMA is a third-order moment algorithm. They are classified as gradient search algorithms, whose robustness can be reflected through the sensitivity of the objective functions to noise [37]. When their robustness is compared, the objective functions of third-order moment algorithms can be uniformly represented by $E({\left|y\right|}^{3})$, and those of fourth-order moment algorithms can be uniformly represented by $E({\left|y\right|}^{4})$ [25].

According to Figure 4, for the output signal $y=u^{T}w$, *u* is the input vector and *w* is coefficient vector of the FIR filter. *u* can be written as u=x+z, *x* represents the signal, and *z* represents the AWGN, with a mean of 0 [25]. Thus, it can be determined that $y={\left(x+z\right)}^{T}w=x^{T}w+z^{T}w=v+d$, where *v* represents the signal; *d* represents the AWGN with a mean of 0, i.e., E(d)=0 and $E(d^{3})=0$; and *v* and *d* are independent of each other [25]. For the PAM-PPM signal, *y* is a real number, so $E\left({\left|y\right|}^{4}\right)=E\left(y^{4}\right)$, and $E\left({\left|y\right|}^{3}\right)=E\left(y^{3}\right)$ when y>0, while $E\left({\left|y\right|}^{3}\right)=-E\left(y^{3}\right)$ when y<0.

$E(y^{3})$ can be expressed as

(28) $$\begin{array}{c} E\left(y^{3}\right)=E\left[{\left(v+d\right)}^{3}\right]=E\left(v^{3}+3v^{2}d+3vd^{2}+d^{3}\right)\\ \begin{array}{c} \;\;\;\;\;\;\;\;\;\;=E\left(v^{3}\right)+E\left(3vd^{2}\right). \end{array} \end{array}$$

$E(y^{4})$ can be expressed as

(29) $$\begin{array}{c} E\left(y^{4}\right)=E\left[{\left(v+d\right)}^{4}\right]=E\left(v^{4}+4v^{3}d+6v^{2}d^{2}+4vd^{3}+d^{4}\right)\\ \begin{array}{c} \;\;\;\;\;\;\;\;\;\;=E\left(v^{4}\right)+E\left(6v^{2}d^{2}\right)+E\left(d^{4}\right). \end{array} \end{array}$$

It can be determined that $E(3vd^{2})$ in Equation (28) and $E\left(6v^{2}d^{2}\right)+E\left(d^{4}\right)$ in Equation (29) represent the error signals induced by noise. Replacing average values with instantaneous values, the error signals of third-order moment algorithms and fourth-order moment algorithms can be represented as $e_{t}=3vd^{2}$ and $e_{f}=6v^{2}d^{2}+d^{4}$, respectively. If we suppose that the variance in *d* is $\sigma_{n}^{2}$, and the pulse amplitudes of *m*PAM-*l*PPM are still 2, 4, 6, …, 2m−2, 2m (m>1), it can be determined that

(30) $$E\left(e_{t}\right)=E\left(3vd^{2}\right)=\frac{3\left(m+1\right)\sigma_{n}^{2}}{l}$$

(31) $$E\left(e_{f}\right)=E\left(6v^{2}d^{2}\right)+E\left(d^{4}\right)=\frac{4\left(m+1\right)\left(2m+1\right)\sigma_{n}^{2}}{l}+3\sigma_{n}^{4}.$$

Because m>1 and l>1, it can be determined that 3(m+1)/l<4(m+1)(2m+1)/l; thus, $E\left(e_{t}\right)<E\left(e_{f}\right)$ with fixed $\sigma_{n}^{2}$. Therefore, the MTOMA is more robust to noise compared with the fourth-order moment algorithms. In this case, the MTOMA can achieve a faster convergence rate and better BER performance [25].

#### 3.2.2. Implementation Complexity

Implementation complexity can be analyzed from the perspectives of time complexity and space complexity. Suppose that the input vector $u\left(n\right)$ and the coefficient vector of the FIR filter $w\left(n\right)$ are both *N*-dimensional vectors.

Time complexity can be analyzed based on the number of basic operations required by the algorithm, including addition, multiplication, exponentiation (also known as multiplication), absolute value calculation, comparison, assignment, etc. [38].

For the MTOMA, the algorithm includes two aspects, including the iterative formula $w\left(n+1\right)=w\left(n\right)+\mu u\left(n\right)y\left(n\right)\left[\left|y\left(n\right)\right|-R\left(n\right)\right]$ (the decision principle of $R\left(n\right)$ is shown in Table 1) and the output signal $y\left(n\right)=u^{T}\left(n\right)w\left(n\right)$. It can be determined that the MTOMA has 7N+4 basic operations, including 4N multiplication operations. Its time complexity is $O\left(N\right)$.

Similarly, the CMA has 7N+2 basic operations, including 4N+1 multiplication operations. The time complexity is $O\left(N\right)$. The MCMA has 7N+6 basic operations, including 4N+2 multiplication operations. The time complexity is also $O\left(N\right)$.

It can be seen that the MTOMA will not increase the time complexity compared with the CMA and MCMA. In addition, the MTOMA requires the fewest multiplication operations, and it is important in practical hardware implementation. For example, a field-programmable gate array (FPGA) utilizes the intellectual property (IP) core to conduct a multiplication operation, which can increase the computation time. Therefore, the MTOMA has faster computation speed for hardware implementation compared with the CMA and MCMA.

Space complexity can be analyzed based on the memory resources required by the algorithms [39]. For the MTOMA, according to the output signal $y\left(n\right)=u^{T}\left(n\right)w\left(n\right)$ and the iterative formula $w\left(n+1\right)=w\left(n\right)+\mu u\left(n\right)y\left(n\right)\left[\left|y\left(n\right)\right|-R\left(n\right)\right]$, $u\left(n\right)$ and $w\left(n\right)$ are *N*-dimensional vectors, and the others are scalars. Thus, the space complexity of the MTOMA is $O\left(N\right)$. Similarly, the space complexity of the CMA and MCMA is also $O\left(N\right)$. Therefore, the MTOMA will not increase the space complexity compared with the CMA and MCMA.

## 4. Simulation Results and Discussions

In this section, we take 4PAM-4PPM as an example to simulate the effects of MTOMA equalization on the *m*PAM-*l*PPM optical signal. For comparison, CMA and MCMA equalization are also simulated. The typical parameters of turbulence and the simulation conditions of the system are presented in Table 2 and Table 3, respectively.

The critical simulation values, which are given in Table 3, are as follows: (1) The laser wavelength λ is set to 1550 nm, because wavelengths around this value are less susceptible to the turbulence effect in engineering [19]. (2) The optical-to-electrical conversion efficiency η is set to 1, because the η of APD can be approximately 1 when the laser wavelength is 1550 nm [26]. (3) The displacement standard deviation $\sigma_{s}$ is set to 0.02 under normalized conditions, because under the control of current acquisition, tracking, and pointing (ATP) systems in FSO communication, the $\sigma_{s}$ of pointing errors is usually limited to 0.02 under normalized conditions [31]. (4) The length of the FIR filter $Len\left(w\right)$ is set to 15, because the equalization algorithms in this case can usually adapt to all turbulence conditions in practical applications [40]. In addition, the initialization $w\left(0\right)$ is set to ${\left[\begin{array}{ccc} 0\cdot \cdot \cdot 0 & 1 & 0\cdot \cdot \cdot 0 \end{array}\right]}^{T}$, which is the standard setting in FSO engineering applications [40]. (5) The step size μ is set to 0.01, which can usually maintain a good balance between convergence rate and BER performance in practical systems [40].

In addition, the signal-to-noise ratio (SNR) in this section is defined as the received SNR in the electrical domain, which is given by $\mathrm{SNR}={\left[\eta E\left(I\right)\right]}^{2}/\sigma_{n}^{2}$, and $\sigma_{n}^{2}$ is the variance of AWGN. Based on this definition, which is also given in Ref. [41], ${\left[\eta E\left(I\right)\right]}^{2}$ can be regarded as the received signal power, and $\sigma_{n}^{2}$ can be regarded as the noise power [41]. Therefore, according to the simulation conditions in Table 3, we can derive E(I)=0.5, so the SNR can be expressed as $\mathrm{SNR}={\left[\eta E\left(I\right)\right]}^{2}/\sigma_{n}^{2}=1/\left(4\sigma_{n}^{2}\right)$.

**Figure 6:**
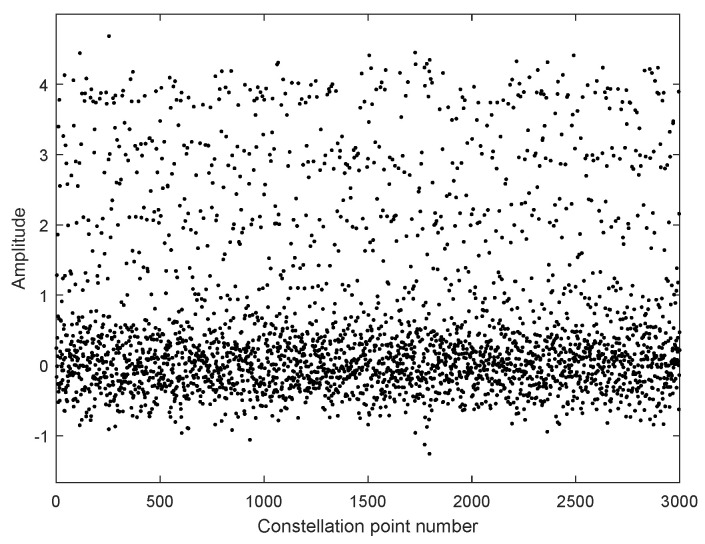
Constellation points of 4PAM-4PPM in atmospheric channel without MTOMA equalization ($\sigma_{R}^{2}=1$; SNR = 15 dB).

**Figure 7:**
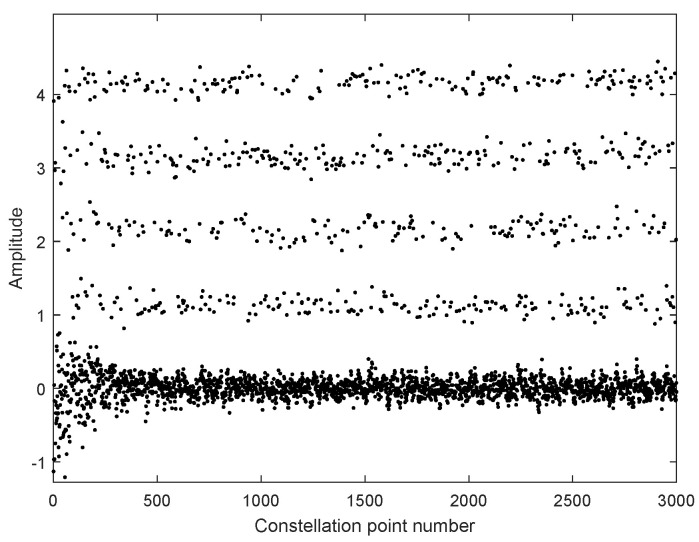
Constellation points of 4PAM-4PPM in atmospheric channel with MTOMA equalization ($\sigma_{R}^{2}=1$; SNR = 15 dB).

In Figure 6, we take moderate turbulence ($\sigma_{R}^{2}=1$) as an example to simulate the constellation points of 4PAM-4PPM in the atmospheric channel. The constellation points are quite scattered under the influence of the turbulence effect. If they are directly used for symbol decision, a high BER is inevitable. Thus, equalization is necessary. The constellation points with MTOMA equalization are presented in Figure 7. It can be seen that, in the early stage of the equalization process, the constellation points converge, and when the MTOMA reaches convergence, they are effectively separated in terms of amplitude. This indicates the effectiveness of MTOMA equalization. In addition, the optical signal suffers from attenuation because of the pointing error, and according to the simulation conditions in Table 3, the signal amplitudes at the receiver are reduced by half compared with those at the transmitter.

**Figure 8:**
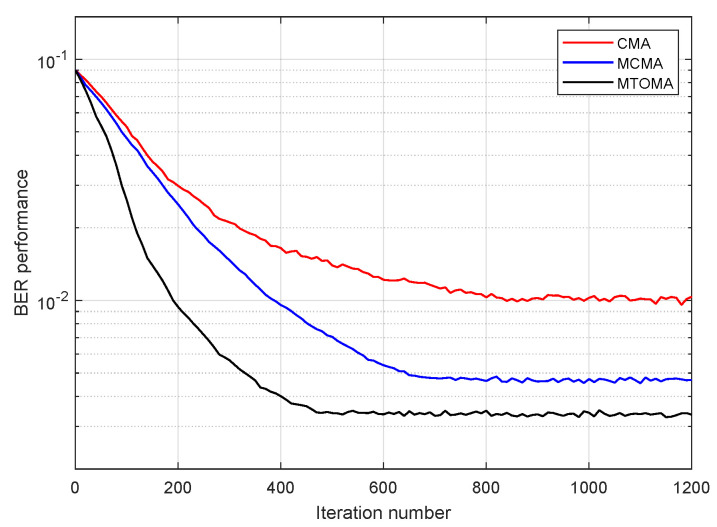
BER performance of 4PAM-4PPM plotted against the iteration number based on CMA, MCMA, and MTOMA equalization ($\sigma_{R}^{2}=1$; SNR = 15 dB).

The BER performance of 4PAM-4PPM based on CMA, MCMA, and MTOMA equalization is presented in Figure 8. Notably, here, “BER performance” represents the BER level of current constellation status in terms of a certain iteration number, rather than the overall BER statistic of the signal. It can be seen that BER performance with the MTOMA is superior to that with the CMA and MCMA under the same iteration number. Thus, BER performance with the MTOMA decreases the fastest in the early stage of the iterative process, and after approximately 530 iterations, it is nearly stable. In contrast, BER performance with the MCMA and CMA is stable when the iteration numbers are about 700 and 750, respectively. Therefore, the MTOMA has a faster convergence rate than the MCMA and CMA when the PAM-PPM signal is processed.

**Figure 9:**
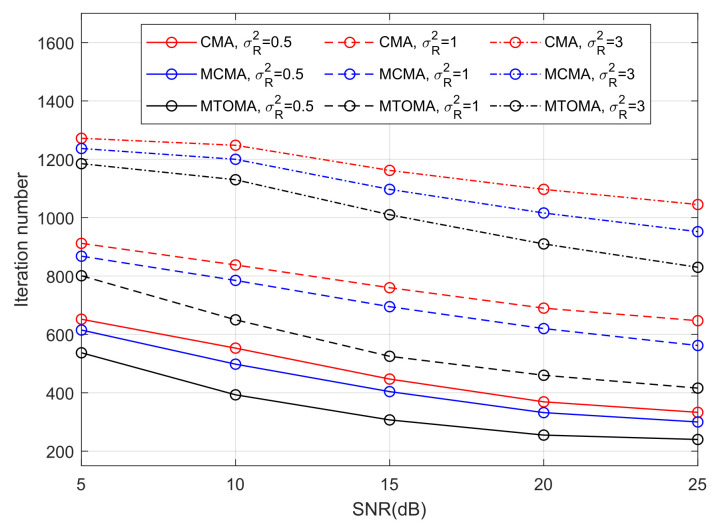
The iteration numbers when algorithms reach convergence under different turbulence conditions.

It can be seen from Figure 9 that turbulence intensity has a significant impact on the convergence rate of algorithms. When the algorithms reach convergence, there are considerably fewer iterations in weak turbulence than in strong turbulence. For example, as the Rytov variance $\sigma_{R}^{2}$ varies from 0.5 (weak turbulence) to 3 (strong turbulence), the iteration numbers of the MTOMA, MCMA, and CMA increase to about 700, 690, and 720, respectively, when SNR = 15 dB. In addition, if SNR and $\sigma_{R}^{2}$ are fixed, the convergence rate of the MTOMA is faster than that of the MCMA and CMA, and the gap is the most obvious in moderate turbulence ($\sigma_{R}^{2}=1$). For example, when SNR = 15 dB, there are about 170 and 230 fewer iterations when using the MTOMA than when using the MCMA and CMA, respectively, in moderate turbulence. In contrast, these values are about 100 and 140 in weak turbulence, and 90 and 150 in strong turbulence.

**Figure 10:**
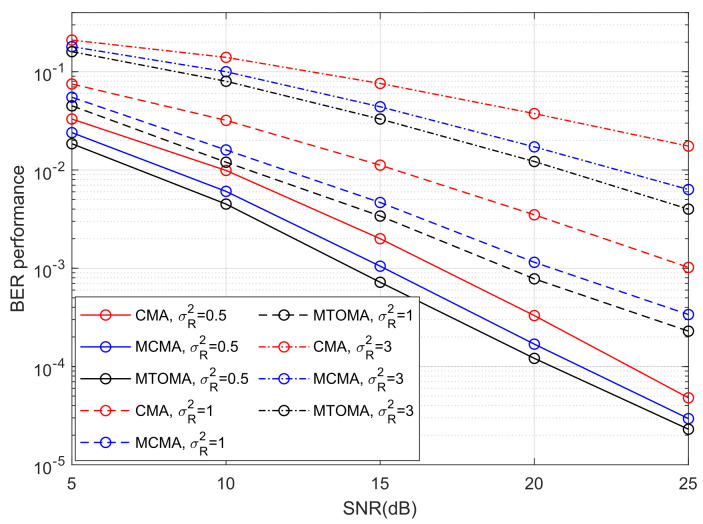
The BER performance of 4PAM-4PPM when algorithms reach convergence under different turbulence conditions.

The BER performance of 4PAM-4PPM under different turbulence conditions when algorithms converge is shown in Figure 10. BER performance can be significantly impacted by turbulence intensity, and it will decrease with an increase in $\sigma_{R}^{2}$. When SNR = 15 dB and $\sigma_{R}^{2}$ varies from 0.5 to 3, BER performance when using the MTOMA, MCMA, and CMA decreases by 16.6 dB, 16.3 dB, and 15.8 dB, respectively. If SNR and $\sigma_{R}^{2}$ are fixed, BER performance when using the MTOMA is superior to that when using the MCMA and CMA, and the gaps between BER performance values are the largest in moderate turbulence. For example, when SNR = 20 dB and $\sigma_{R}^{2}=1$, the BER performance gaps are 1.7 dB and 6.5 dB, respectively, between the MTOMA and the other two algorithms. In contrast, the gaps are 1.4 dB and 4.3 dB when $\sigma_{R}^{2}=0.5$, and 1.5 dB and 4.8 dB when $\sigma_{R}^{2}=3$.

Furthermore, the results of the performance comparison between the MTOMA and fourth-order moment algorithms are summarized in Table 4, Table 5, Table 6 and Table 7 according to Figure 9 and Figure 10.

**Figure 11:**
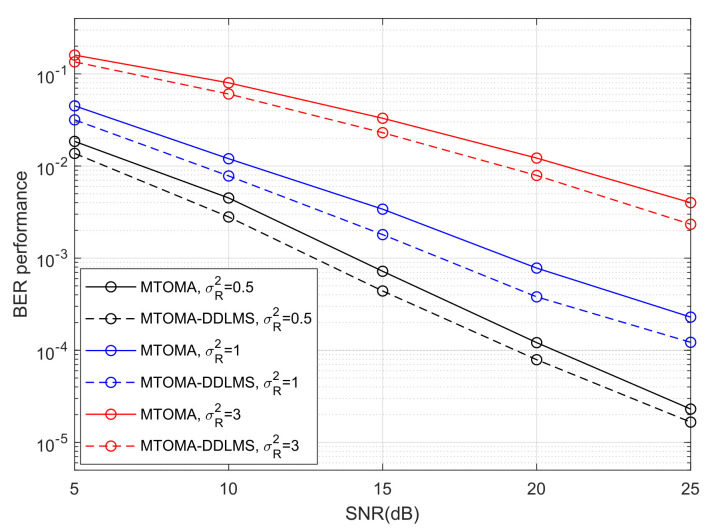
The BER performance of 4PAM-4PPM when the MTOMA and MTOMA-DDLMS reach convergence under different turbulence conditions.

Figure 9 shows the iteration numbers when the MTOMA reaches convergence, according to which we can continue to utilize the DDLMS, and BER performance is presented in Figure 11. When SNR and $\sigma_{R}^{2}$ are fixed, BER performance with the MTOMA-DDLMS is superior to that with the MTOMA under different turbulence conditions. Therefore, the dual-mode equalization algorithm MTOMA-DDLMS further improves the BER performance of the PAM-PPM signal compared with the MTOMA. In addition, the effect of the MTOMA-DDLMS is the most significant under moderate turbulence conditions. For example, when SNR = 15 dB, BER performance with the MTOMA-DDLMS can increase by 2.8 dB compared with the MTOMA in moderate turbulence. By comparison, the BER performance gains are 2.1 dB and 1.6 dB, respectively, in weak and strong turbulence. When SNR = 20 dB, the BER performance gains are 1.8 dB, 3.1 dB, and 1.9 dB, respectively, in weak, moderate, and strong turbulence.

**Figure 12:**
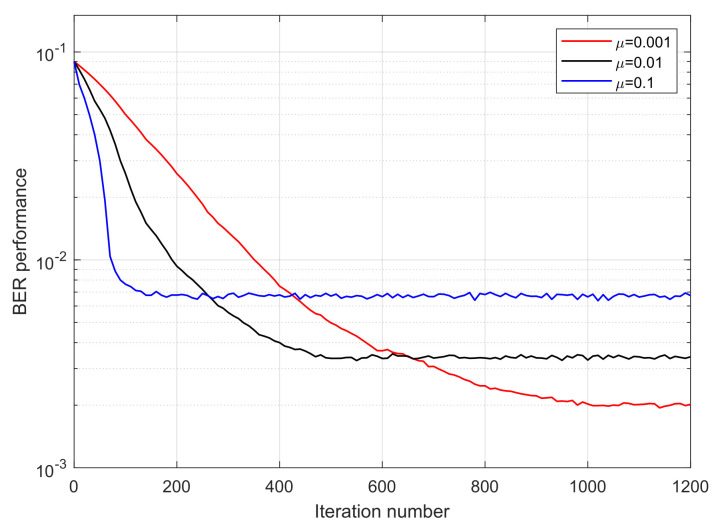
BER performance of 4PAM-4PPM plotted against the iteration number based on the MTOMA with different step sizes ($\sigma_{R}^{2}=1$; SNR = 15 dB).

**Figure 13:**
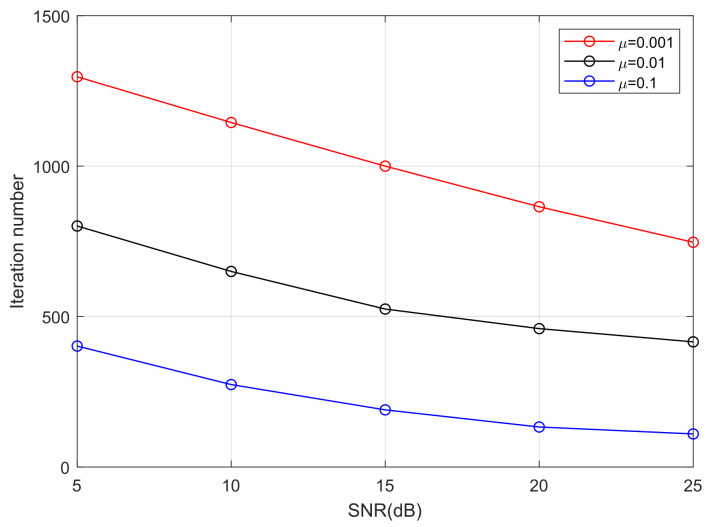
The iteration numbers when the MTOMA reaches convergence based on 4PAM-4PPM with different step sizes ($\sigma_{R}^{2}=1$).

**Figure 14:**
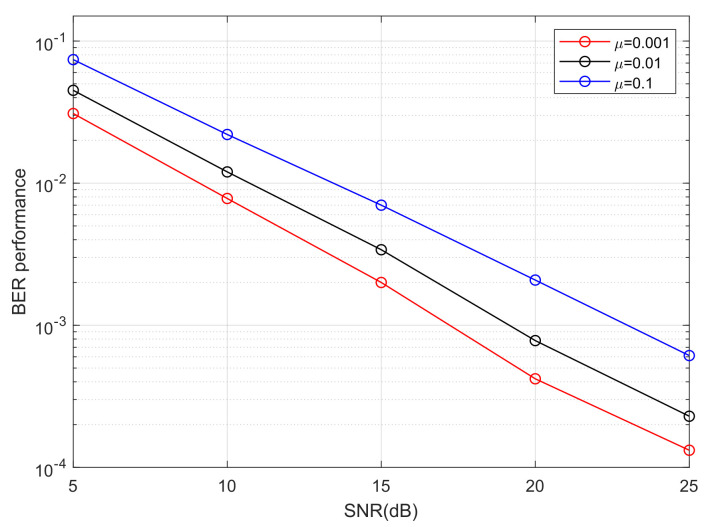
The BER performance of 4PAM-4PPM when the MTOMA reaches convergence with different step sizes ($\sigma_{R}^{2}=1$).

We take moderate turbulence ($\sigma_{R}^{2}=1$) as an example to analyze the influence of step size on PAM-PPM optical signals in an atmospheric turbulence channel. Figure 12 and Figure 13 demonstrate that step size has a significant impact on the convergence rate of the MTOMA. When SNR is fixed, as the step size increases, the convergence rate will increase; however, according to Figure 14, BER performance under convergence will decrease, and different application scenarios may require different step sizes.

For real-time data transmission scenarios, such as voice calls, a fast convergence rate is required to reduce call drops and delays [42]. In addition, when the channel conditions change frequently, a fast convergence rate is also required to rapidly adapt to the channel variation [43]. For example, in military applications, when unmanned aerial vehicles (UAVs) are constantly moving, UAV-based FSO systems must quickly track channel variation to keep transmission links stable [44]. Moreover, when FSO communication is used in a marine environment, a fast convergence rate is also necessary because atmospheric turbulence can change frequently [45]. In these situations, large step sizes tend to be required. For transmission scenarios with stable channel conditions, such as high-definition (HD) video transmission between satellites and the ground, and television signal transmission in cities, high BER performance may be the most important factor [46]. In these situations, small step sizes tend to be required.

## 5. Conclusions

In this paper, a blind equalization scheme based on the MTOMA is proposed to mitigate the influence of the turbulence effect on PAM-PPM optical signals, which represents a promising avenue for future high-speed FSO communication. Through theoretical analysis, it is found that the MTOMA is more robust to noise compared with the current fourth-order moment algorithms, such as the CMA and MCMA. Meanwhile, the MTOMA will not increase the implementation complexity compared with the CMA and MCMA; in particular, it has fewer multiplication operations, which is crucial, because it indicates that the MTOMA has faster computation speed for hardware implementation.

These simulation results show that the MTOMA is effective in reducing the distortion of PAM-PPM optical signals in atmospheric turbulence channels with pointing errors. Under different turbulence conditions, the MTOMA has a faster convergence rate than the CMA and MCMA. For example, when SNR is 15 dB, the MTOMA requires about 530 iterations to reach convergence in moderate turbulence, which is about 230 and 170 fewer iterations than required by the CMA and MCMA, respectively; the differences in the number of iterations required by the MTOMA and those required by the CMA and MCMA, respectively, are about 140 and 100 in weak turbulence and 150 and 90 in strong turbulence. Moreover, when algorithms converge, the BER performance of the PAM-PPM signals with MTOMA is also superior to that with CMA and MCMA. For example, when SNR = 20 dB, the BER performance of the PAM-PPM signals with MTOMA improves by 6.5 dB and 1.7 dB, respectively, compared to that with CMA and MCMA in moderate turbulence, and this value improves by 4.3 dB and 1.4 dB in weak turbulence and 4.8 dB and 1.5 dB in strong turbulence. Therefore, the reliability of the PAM-PPM optical system with the MTOMA can be enhanced. In addition, the dual-mode algorithm MTOMA-DDLMS further improves BER performance compared with the MTOMA.

The current study is based on theoretical analysis and simulation, and some assumptions are made in channel modeling. In the near future, real-world FSO experiments will be conducted to further verify the feasibility of the MTOMA in PAM-PPM optical systems, and the proposed scheme will be continuously enhanced in practical experiments. Meanwhile, future research may include the following aspects. Firstly, a variable step size scheme can be combined with the MTOMA to improve flexibility and adaptability in time-varying channels. Secondly, channel coding technology, such as current Polar codes and LDPC codes, can be combined with the MTOMA to further improve the BER performance of PAM-PPM optical systems in strong turbulence conditions. Thirdly, the MTOMA may be extended to other asymmetric signals with high spectral efficiency, such as polarization–division–multiplexed PAM-PPM (PDM-PAM-PPM).

## Figures and Tables

**Table 1:** Variation principle of $R\left(n\right)$ (k=1,2,…,m−1).

Value of $y\left(n\right)$	$R\left(n\right)$ Decision
$y\left(n\right)\leq \eta E\left(I\right)$	$R\left(n\right)=0$
(2k−1)ηE(I)<y(n)≤(2k+1)ηE(I)	$R\left(n\right)=2k\eta E\left(I\right)$
$y\left(n\right)>\left(2m-1\right)\eta E\left(I\right)$	$R\left(n\right)=2m\eta E\left(I\right)$

**Table 2:** Typical parameters of turbulence.

Turbulence Conditions	$\sigma_{R}^{2}$	α	β
Weak turbulence	0.5	5.97	4.39
Moderate turbulence	1	4.39	2.56
Strong turbulence	3	4.12	1.43

**Table 3:** Simulation conditions of the system.

Parameter	Symbol	Value
Communication rate	$R_{b}$	1 Gbit/s
Wavelength of laser	λ	1550 nm
Transmission distance	*z*	15 km
Optical-to-electrical conversion efficiency	η	1
Beam radius at transmitter	$w_{0}$	0.1 m
Radius of detector	$R_{a}$	0.1 m
Displacement standard deviation	$\sigma_{s}$	0.02
Length of FIR filter	$Len\left(w\right)$	15
Initialization of FIR	$w\left(0\right)$	${\left[\begin{array}{ccc} 0\cdot \cdot \cdot 0 & 1 & 0\cdot \cdot \cdot 0 \end{array}\right]}^{T}$
Step size	μ	0.01

**Table 4:** Performance comparison with different algorithms (SNR = 10 dB).

Performance Comparison	Turbulence Condition	MTOMA	CMA	MCMA
	$\sigma_{R}^{2}=0.5$, weak	390	550	500
Iteration numbers	$\sigma_{R}^{2}=1$, moderate	650	840	790
	$\sigma_{R}^{2}=3$, strong	1130	1250	1200
	$\sigma_{R}^{2}=0.5$, weak	$4.5\times 10^{-3}$	$9.9\times 10^{-3}$	$6.1\times 10^{-3}$
BER performance	$\sigma_{R}^{2}=1$, moderate	$1.2\times 10^{-2}$	$3.2\times 10^{-2}$	$1.6\times 10^{-2}$
	$\sigma_{R}^{2}=3$, strong	$8\times 10^{-2}$	$1.4\times 10^{-1}$	$1\times 10^{-1}$

**Table 5:** Performance comparison with different algorithms (SNR = 15 dB).

Performance Comparison	Turbulence Condition	MTOMA	CMA	MCMA
	$\sigma_{R}^{2}=0.5$, weak	310	450	410
Iteration numbers	$\sigma_{R}^{2}=1$, moderate	530	760	700
	$\sigma_{R}^{2}=3$, strong	1010	1160	1100
	$\sigma_{R}^{2}=0.5$, weak	$7.2\times 10^{-4}$	$2\times 10^{-3}$	$1.1\times 10^{-3}$
BER performance	$\sigma_{R}^{2}=1$, moderate	$3.4\times 10^{-3}$	$1.1\times 10^{-2}$	$4.7\times 10^{-3}$
	$\sigma_{R}^{2}=3$, strong	$3.3\times 10^{-2}$	$7.6\times 10^{-2}$	$4.4\times 10^{-2}$

**Table 6:** Performance comparison with different algorithms (SNR = 20 dB).

Performance Comparison	Turbulence Condition	MTOMA	CMA	MCMA
	$\sigma_{R}^{2}=0.5$, weak	260	370	330
Iteration numbers	$\sigma_{R}^{2}=1$, moderate	460	690	620
	$\sigma_{R}^{2}=3$, strong	910	1100	1020
	$\sigma_{R}^{2}=0.5$, weak	$1.2\times 10^{-4}$	$3.3\times 10^{-4}$	$1.7\times 10^{-4}$
BER performance	$\sigma_{R}^{2}=1$, moderate	$7.8\times 10^{-4}$	$3.5\times 10^{-3}$	$1.2\times 10^{-3}$
	$\sigma_{R}^{2}=3$, strong	$1.2\times 10^{-2}$	$3.8\times 10^{-2}$	$1.7\times 10^{-2}$

**Table 7:** Performance comparison with different algorithms (SNR = 25 dB).

Performance Comparison	Turbulence Condition	MTOMA	CMA	MCMA
	$\sigma_{R}^{2}=0.5$, weak	240	330	300
Iteration numbers	$\sigma_{R}^{2}=1$, moderate	420	650	560
	$\sigma_{R}^{2}=3$, strong	830	1050	950
	$\sigma_{R}^{2}=0.5$, weak	$2.3\times 10^{-5}$	$4.8\times 10^{-5}$	$3\times 10^{-5}$
BER performance	$\sigma_{R}^{2}=1$, moderate	$2.3\times 10^{-4}$	$1\times 10^{-3}$	$3.4\times 10^{-4}$
	$\sigma_{R}^{2}=3$, strong	$4\times 10^{-3}$	$1.8\times 10^{-2}$	$6.3\times 10^{-3}$

## Data Availability

Data will be made available on request.

## Abbreviations

Abbreviation	Full Name
APD	Avalanche Photon Diode
AWGN	Additive White Gaussian Noise
BER	Bit Error Rate
CDF	Cumulative Distribution Function
CMA	Constant Modulus Algorithm
DAC	Digital-to-Analog Converter
DDLMS	Decision-Directed Least Mean Square
FIR	Finite Impulse Response
FPGA	Field-Programmable Gate Array
FSO	Free-space Optical (Communication)
IM	Intensity Modulator
IM-DD	Intensity Modulation–Direct Detection
MCMA	Modified Constant Modulus Algorithm
MMSE	Minimum Mean Square Error
MTOMA	Modified Third-order Moment Algorithm
OOK	On–Off Keying
PAM-PPM	Pulse Amplitude Modulation–Pulse Position Modulation
PDF	Probability Density Function
PPM	Pulse Position Modulation
P/S	Parallel-to-Serial
SIM	Subcarrier Intensity Modulation
SNR	Signal-to-Noise Ratio
S/P	Serial-to-Parallel
TOMA	Third-order Moment Algorithm
UAV	Unmanned Aerial Vehicle
